# The relationship between mobile phone dependence, self-control, and Tai Chi exercise among sub-health older adults in urban areas: a latent profile analysis

**DOI:** 10.3389/fpubh.2026.1759896

**Published:** 2026-02-11

**Authors:** Tongtong Hao, Dong Wu

**Affiliations:** College of Chinese Wushu, Beijing Sport University, Beijing, China

**Keywords:** older adults, mobile phone dependency, regression analysis, self-control, sub-health, Tai Chi

## Abstract

**Background:**

The study explores the interconnection between the latent categories of mobile phone dependency and self-control in the sub-healthy urban older adults practicing Tai Chi. The findings aim to provide a reference for preventing mobile phone dependence, enhancing self-control and improving sub-health status in this population.

**Methods:**

A multi-stage cluster sampling method was employed to screen 560 sub-healthy urban older adults from 2,946 valid survey responses in Xuzhou City, Jiangsu Province. Sub-health status was verified using the SHMS V1.0 scale. Data were collected between September and October 2025. Latent profile analysis (LPA) was used to categorize mobile phone dependency and self-control. Pearson correlation analysis measured the relationship between these two variables. Additionally, chi-square test examined demographic differences across the identified latent profiles. Finally, multivariate logistic regression analyzed the associations between mobile phone dependency, self-control, and Tai Chi exercise.

**Results:**

LPA identified four distinct profiles: Low dependency-Medium control (109 individuals, 19.5%), High dependency-No control (207 individuals, 37.0%), No dependency-High control (191 individuals, 34.1%), and Moderate dependency-Low control (53 individuals, 9.5%). These categories had statistically significant differences (*p* < 0.05). These were gender, age, ethnicity, marital status, educational attainment, and monthly income. Logistic regression controlled for demographic variables. The low dependence-medium control group served as the reference. Tai Chi exercise exhibited negative correlations with medium dependence-low control group (OR = 2.170). It was also associated with the high dependence-no control group (OR = 1.846). Notably, Tai Chi showed a strong positive association with the no dependence-high control group (OR = 111.599) (all *p* < 0.01).

**Conclusion:**

Tai Chi exercise exerts differential effects on urban sub-healthy older adults across distinct latent profiles of mobile phone dependency and self-control. Societal stakeholders should strengthen Tai Chi programs for these diverse categories to promote their physical and mental wellbeing.

## 1 Introduction

Rapid digitalization and population aging have made health management for urban sub-healthy older adults a critical priority (1, 2). As per the 56th Statistical Report on Internet Development of China issued in July 2025, China has now conquered 161 million internet users of 60 years and above. This represents a penetration rate of 52.04%. Mobile Phone Dependence (MPD) is becoming pervasive within this demographic (3). MPD entails a loss of behavioral control resulting from prolonged, excessive usage. It triggers intense cravings and subsequently impairs social function. Sub-healthy older adults already face physiological and psychological decline. Therefore, they are particularly vulnerable to this negative cycle of MPD (4).

Sub-health in older adults is a transitional “third state” between health and disease (5). It is characterized by a significant decline in physiological function, mental health, and social adaptation, yet it does not meet the clinical diagnostic criteria for a specific illness (6). Epidemiological data shows that sub-health is highly prevalent among China’s urban older adults, with approximately 55–70% of people aged 60 and older experiencing various sub-health symptoms (7). Regarding diagnostic standards, the Sub-Health Measurement Scale (SHMS V1.0) is a widely used tool for standardized assessment (8). This scale effectively identifies issues such as physical fatigue, sleep disturbances, and cognitive decline (9). For the urban sub-healthy older adults, the combination of sedentary lifestyles and the complexities of digital mobile phone use has created an urgent need for health management (10). Targeted interventions are necessary to prevent the deterioration of physical, mental, and social functions into diagnosable clinical diseases.

Self-control (SC) is the fundamental ability to regulate individual behavior with societal expectations. Generally, SC exhibits a negative correlation with MPD at the trait level (11). However, this relationship is not merely additive. It creates a vicious cycle of “resource depletion, diminished control, intensified dependence.” Individuals expend self-control resources to resist digital temptation. Once these resources are exhausted (12). Existing research predominantly focuses on linear correlations. This approach ignores intra-population heterogeneity. Significant disparities likely exist in how MPD and SC combine within different older cohorts. Identifying these variations is essential for understanding their Tai Chi exercise and health behaviors.

Tai Chi serves as a traditional Chinese health-preserving exercise with distinct advantages for sub-healthy older adults (13, 14). Physiologically, its gentle movements enhance muscle strength, flexibility and balance. It also effectively relieves physical fatigue (15, 16). Psychologically, the discipline focusing on breath and movement helps clear distracting thoughts. This fosters emotional stability and psychological resilience (17, 18). Socially, Tai Chi communities provide vital support networks (19, 20). Previous studies suggest links between physical activity, SC, and MPD. Generally, high SC motivates activity, while high MPD reduces it. However, these variable-centered studies fail to capture subtype differences within the “MPD-SC” dimension (21).

LPA serves as an individual-centered analytical method to address this gap. It identifies latent subgroups with similar patterns, overcoming the limitations of variable-centered approaches (22). To our knowledge, no previous study has applied LPA to categorize MPD and SC among sub-healthy urban older adults. Nor have studies examined the specific association with Tai Chi. The study offers three key innovations. First, it uses LPA to uncover heterogeneous subtypes. This addresses the previous neglect of intra-population variation. Second, it quantifies differences in Tai Chi participation across these distinct subtypes. This supports the development of precision intervention strategies. Third, it focuses on the unique interactions between MPD, SC, and health behaviors in this vulnerable population. Therefore, the findings hold significant practical value.

In summary, this study utilizes LPA to dissect latent categories of MPD and SC among sub-healthy urban older adults. It further explores the relationship between these categories and Tai Chi exercise. This not only enriches theoretical research on MPD, SC, and health behaviors. It also provides actionable evidence for intervention programs. Key goals include preventing MPD, enhancing SC, and promoting Tai Chi. Ultimately, this research aids in improving the quality of life for this population and facilitating healthy ageing.

## 2 Methods

### 2.1 Subjects

A multistage cluster sampling method was employed in Xuzhou City, Jiangsu Province, between September and October 2025. To improve transparency, the four-stage sampling process and corresponding numerical details are summarized below:

Stage 1: Selection of Primary Sampling Units (PSUs). Out of the 5 urban districts under Xuzhou’s jurisdiction, 3 districts (Yunlong, Gulou, and Quanshan) were randomly selected using probability proportional to size (PPS) sampling based on the older population.

Stage 2: Selection of Secondary Sampling Units (SSUs). Within the selected districts, 12 sub-districts (4 per district) were randomly selected using simple random sampling from the total pool of available sub-districts.

Stage 3: Determination of Final Survey Points. A total of 24 fixed Tai Chi practice locations (2 per sub-district), including community parks, squares, and activity centers, were identified through field visits.

Stage 4: Systematic Sampling and Subject Screening. Intercept surveys were conducted at these sites, yielding 2,946 valid questionnaire responses. Participants were then screened using the Sub-Health Measurement Scale Version 1.0 (SHMS V1.0) to identify those in a sub-health state. Only the 560 individuals (19.0% of the valid respondents) who met the specific sub-health criteria (total score <60% of the maximum possible score) were included in the final LPA and regression analysis (8).

### 2.2 Sample size

Sample size calculation followed established guidelines for Latent Profile Analysis (LPA) (23). A minimum of 500 participants is suggested in the literature to guarantee sufficient statistical power. To account for a potential 10% invalid response rate, the minimum recruitment requirement was fixed at 550 cases. In this study, a total of 3,537 questionnaires were distributed. We excluded 591 invalid responses (403 samples with identical answers and 188 samples with contradictory responses). Therefore, 2,946 valid questionnaires were obtained, yielding a valid response rate of 83.30%. From this initial pool of 2,946 respondents, 560 individuals were identified as being in a sub-health state according to the SHMS V1.0 scoring criteria and were included as the final study population for analysis. The demographic characteristics and detailed profile distributions reported in the Results section are based strictly on these 560 sub-healthy participants.

**Figure 1:**
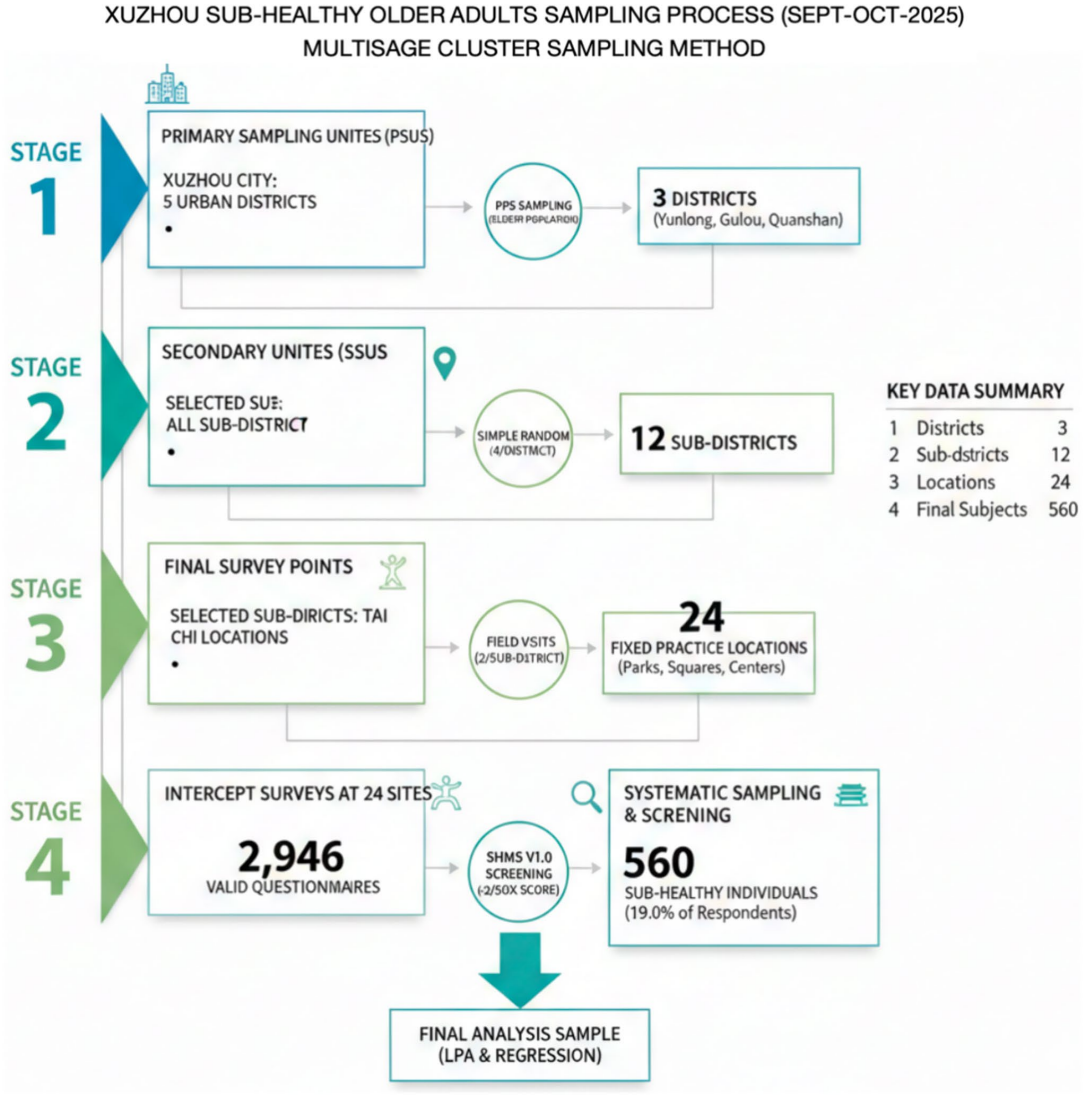
Sampling flowchart.

Informed consent forms were signed by all participants. The Ethics Committee of Beijing Sport University approved the study (Approval No. 2025347H) (Figure 1).

### 2.3 Research instruments

#### 2.3.1 Older adults mobile phone dependence scale

We employed a revised version of the Mobile Phone Addiction Index (MPAI) by Leung (24). Originally designed for adolescents, specific items were modified to reflect the study objectives. For instance, the phrase “friends and family” was updated to “children, partner, and friends.” Similarly, the item regarding anxiety about missing messages was expanded to include children. The Mobile Phone Addiction Inventory (MPAI) comprises 17 items across four dimensions, including loss of control, withdrawal, escapism, and inefficiency. It has a five-point Likert scale (1 = never to 5 = always). Total scores range from 17 to 85. Higher scores indicate greater dependency. Respondents scoring 4 or higher on eight or more items are classified as having dependency behavior (25). The total Cronbach’s *α* for this survey was 0.829. Adjusted item-total correlations for the four dimensions—loss of control, withdrawal, escapism, and inefficiency—were 0.650, 0.695, 0.662, and 0.618, respectively. These values significantly exceed the 0.4 threshold. This indicates strong overall reliability for the scale items.

To further evaluate the convergent validity of the adapted Older Adults Mobile Phone Dependence Scale (OAMPDS), Composite Reliability (CR) and Average Variance Extracted (AVE) were calculated for its four dimensions. The CR values ranged from 0.854 to 0.950, and the AVE values ranged from 0.546 to 0.793. All indices exceeded the recommended thresholds of 0.7 and 0.5, respectively. These results indicate robust convergent validity and internal consistency for the scale within this sub-healthy older adult study population.

#### 2.3.2 Self-control scale

The Self-Control Scale created by Tangney et al. (26) and revised by Tan and Guo (27). This instrument comprises 19 items across five dimensions. These include impulse control, healthy habits, moderation in leisure, work focus, and temptation resistance. The scale utilizes a five-point Likert scale ranging from 1 (strongly disagree) to 5 (strongly agree). Total scores range from 19 to 95. Higher scores denote greater self-control. Previous research shows the scale’s reliability and validity in older adults (28). In this survey, the total Cronbach’s *α* for the scale was 0.961.

#### 2.3.3 Tai Chi exercise level scale

The Tai Chi exercise volume was measured using a scale revised from Liang’s Physical Activity Rating Scale-3 (PARS-3) (29). Considering the physiological characteristics of the older adults, the intensity dimension was adjusted to reflect perceived exertion rather than absolute physical load, which is more appropriate for assessing exercise in older populations. Intensity was categorized into five levels based on subjective sensations: (1) Very light (nearly no change in breathing), (2) Light (slight increase in heart rate), (3) Moderate (slight breathlessness but able to talk comfortably), (4) Large (heavy breathing), and (5) Very large (near exhaustion). A sample item for intensity is: “When practicing Tai Chi, to what degree do you experience changes in your breathing or heart rate?” The physical activity volume was calculated as: intensity × (duration - 1) × frequency. The scale demonstrated excellent internal consistency (Cronbach’s *α* = 0.913). To further evaluate the convergent validity of the Tai Chi Exercise Level Scale, Composite Reliability (CR) and Average Variance Extracted (AVE) were calculated. The CR was 0.887 and the AVE was 0.724, both exceeding the recommended thresholds of 0.7 and 0.5, respectively. These results indicate robust convergent validity and internal consistency for the scale in this study population.

#### 2.3.4 Sub-health measurement scale version 1.0

The Sub-Health Measurement Scale Version 1.0 (SHMS V1.0) was used to screen participants. The scale has demonstrated good psychometric properties in Chinese populations, with a sensitivity of 0.85 and a specificity of 0.88 (30). In this study, sub-health status was determined using a cut-off value based on the total score, where individuals scoring less than 60% of the maximum possible total score were categorized as being in a sub-health state (31).

To ensure the quality of data collection, all assessors (research assistants) underwent a 3-day standardized training program. This training included a detailed explanation of each item in the scale, simulated interview exercises, and consistency testing to ensure standardized administration and minimize inter-rater bias (32).

### 2.4 Quality control

Prior to data collection, survey staff received systematic training. This ensured a clear understanding of the questionnaire structure and response protocols. Regarding online distribution, we specified response requirements and target demographics in advance. For offline surveys, we relied on the cooperation of older participants to facilitate distribution. The entire process remained anonymous. Questionnaires were distributed and collected on-site. Subsequently, data underwent double-entry verification (33). The whole process was anonymous, with questionnaires being distributed and collected on-site. Invalid questionnaires with missing values or arbitrary responses were excluded.

### 2.5 Statistical analysis

Latent profile analysis was performed using Mplus 8.3. Model optimization relied on: Akaike Information Criterion (AIC), Bayesian Information Criterion (BIC), and sample size-adjusted BIC (aBIC), where lower values represent better model fit (34). *p* < 0.05 in LMR and BLRT tests signified model improvement. Entropy values >0.80 signified high classification accuracy (35). Data analysis employed SPSS 27.0 software. Categorical data were analyzed via frequencies and percentages. Intergroup comparisons used chi-square tests. Normality testing confirmed that dependency and self-control scores followed normal distributions. Pearson correlation evaluated the relationship between these variables. Multiple logistic regressions examined the effect of Tai Chi exercise on the latent categories. Tai Chi scores were standardized using Z-scores to reduce the impact of extreme values. The level of significance was established to be 0.05.

Moreover, Multicollinearity diagnostics were conducted prior to the multivariate logistic regression. The Variance Inflation Factor (VIF) values for all independent variables were between 1.05 and 2.43, well below the threshold of 5, indicating no significant multicollinearity among the predictors. To mitigate potential estimation bias and instability arising from small subgroup sizes (e.g., Category C4, *n* = 53) and extreme odds ratios, Firth’s penalized likelihood regression was employed. This method provides more reliable estimates and narrower confidence intervals when dealing with sparse data in specific latent profiles.

## 3 Results

### 3.1 Correlation analysis of mobile phone dependence and self-control in sub-healthy urban older adults

Prior to conducting Pearson correlation analysis, a Shapiro–Wilk test was performed to examine the normality of the data. The results indicated that all key variables followed a normal distribution (*W* > 0.90, *p* > 0.05), justifying the use of parametric tests for subsequent analyses.

The study included 560 sub-healthy urban older adults. The detection rate for mobile phone dependency was 19.0%. The sample comprised 279 males and 281 females. Age distribution included 168 individuals aged 60–69 years (younger older adults), 206 aged 70–79 years (middle-aged older adults), and 186 aged 80 years and above (older adults). Correlation analysis revealed a consistent pattern. All four dimensions of mobile phone dependency correlated negatively with the five dimensions of self-control (*p* < 0.01). A comprehensive description of the results is in Table 1.

**Table 1:** Correlation analysis between mobile phone dependency and self-control scale scores among sub-healthy older adults in urban areas (*r*-values, *n* = 560).

Variable	Mean	Standard deviation	1	2	3	4	5	6	7	8	9
1. Withdrawal	2.931	1.087	–								
2. Uncontrollability	2.838	1.002	0.591**	–							
3. Avoidance	2.862	1.044	0.584**	0.545**	–						
4. Inefficiency	2.827	1.005	0.547**	0.494**	0.526**	–					
5. Impulse control	3.031	1.093	−0.632**	−0.514**	−0.526**	−0.472**	–				
6. Health habits	3.074	1.203	−0.561**	−0.541**	−0.518**	−0.521**	0.566**	–			
7. Resisting temptation	3.099	1.2	−0.600**	−0.572**	−0.547**	−0.509**	0.552**	0.521**	–		
8. Focus on work	3.102	1.164	−0.566**	−0.516**	−0.547**	−0.540**	0.479**	0.543**	0.493**	–	
9. Restricting entertainment	3.069	1.162	−0.506**	−0.505**	−0.505**	−0.495**	0.491**	0.475**	0.526**	0.535**	–

*p < 0.05 **p < 0.01.

### 3.2 Validity and reliability analysis

The EFA results yielded a four-factor structure consistent with the original scale, explaining 82.824% of the total variance. The KMO value was 0.947, and Bartlett’s test of sphericity was significant (*p* < 0.001), indicating excellent suitability for factor analysis (Table 2). In the CFA, the model demonstrated robust psychometric properties. The Composite Reliability (CR) for the four dimensions ranged from 0.854 to 0.950, and the Average Variance Extracted (AVE) ranged from 0.546 to 0.793, all exceeding the recommended thresholds of 0.7 and 0.5, respectively (Table 3). These results confirm strong convergent validity. Furthermore, the Heterotrait-Monotrait Ratio (HTMT) values were within acceptable ranges, supporting the discriminant validity of the adapted instrument.

**Table 2:** KMO and Bartlett’s test of sphericity for the adapted OAMPDS.

KMO measure of sampling adequacy		0.947
Bartlett’s test of sphericity	χ^2^	9078.957
	*df*	136
	*p*	0.000

**Table 3:** Average variance extracted (AVE) and composite reliability (CR) for the adapted OAMPDS dimensions.

Factor	AVE	CR
Loss of control	0.793	0.950
Withdrawal-related	0.546	0.854
Escapism	0.645	0.876
Inefficiency	0.718	0.884

### 3.3 Latent profile analysis of mobile phone dependence and self-control among sub-healthy urban older adults

Latent profile analysis employed mean scores from the four dimensions of the OAMPDS and five dimensions of the SCS as indicators. Five latent profile models (from 1-class to 5-class) were sequentially defined and subjected to model fit analysis. As shown in Table 4, although the AIC, BIC, and aBIC continued to decrease slightly as the number of profiles increased, the magnitude of the reduction diminished significantly after the four-class model. Specifically, while the LMR and BLRT criteria showed satisfactory model fit for the 2-class to 4-class models (*p* < 0.01), the LMR test for the five-class model was no longer statistically significant (*p* = 0.156). This indicates that the inclusion of a fifth latent profile did not yield a significant improvement in model fit over the four-class solution. Furthermore, the four-class model exhibited the highest Entropy value (0.875), suggesting superior classification precision compared to other models. The mean probability of classification for individuals in each group was above 88%, indicating robust model reliability. Considering statistical parsimony, the distinctiveness of the groups, and the theoretical interpretability of the profiles, the four-category classification (C1, C2, C3, C4) was identified as the optimal model. Under this four-class structure, the resulting distributions were 19.5, 37.0, 34.1, and 9.5%.

**Table 4:** Model fit indices for latent profile models of mobile phone dependency and self-control among older adults.

Class	AIC	BIC	aBIC	LMR (p)	BLRT (p)	Entropy	Grouping situation
1 class	15328.445	15,406.348	15349.207	–	–	–	560
2 class	12852.801	12,973.983	12,885.097	0.000	0.000	0.832	206, 354
3 class	12592.516	12756.977	12636.347	0.000	0.000	0.867	192, 210, 158
4 class	12565.221	12772.962	12620.586	0.001	0.000	0.875	109, 207, 191, 53
5 class	12558.110	12790.340	12625.420	0.156	0.000	0.852	102, 191, 179, 50, 38

**Figure 2:**
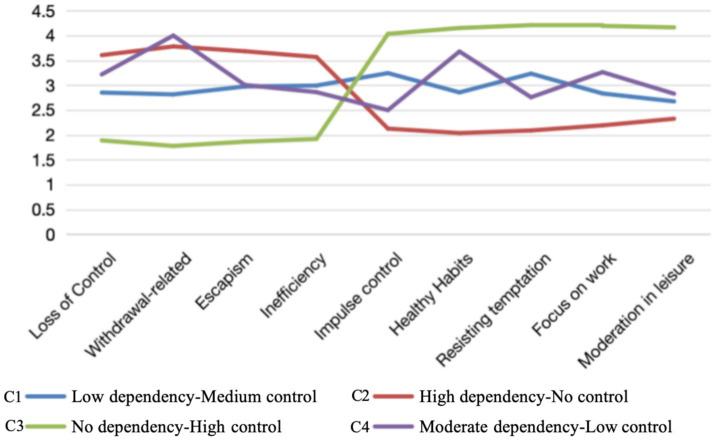
Scores of MPAI and SCS dimensions for latent categories of mobile phone dependence and self-control in older adults.

The four latent categories showed significant differences across the four OAMPDS dimensions and five SCS dimensions. This indicates the presence of notable group characteristics. As illustrated in Figure 2, Category C1 scored lower than C2 and C4 but higher than C3 on items 1–4 (mobile phone dependency dimension). On items 5–9 (self-control dimension), it scored higher than C2 but lower than C3. This group comprised 19.5% (109 individuals) of participants and is termed the “Low dependency-Medium control type.” Category C2 scored markedly higher than C1 and C3 on items 1–4, yet significantly lower than C1, C3, and C4 on items 5–9. Accounting for roughly 37.0% (207 individuals), this category is termed the “High dependency-No control type.” Category C3 scored significantly lower than C1, C2, and C4 on items 1–4. Conversely, it scored significantly higher than the other three categories on items 6–9. This category comprised approximately 34.1% (191 individuals) of all participants and was designated as the “No dependency-High control.” Category C4 scored significantly higher than C3 on items 1–4, and higher than C2 but lower than C3 on items 5–9, comprising approximately 9.5% (53 individuals) of all participants. Based on these characteristics, it is classified as the “Moderate dependency-Low control” type.

The detailed definitions and interpretations of each latent profile are summarized in Table 5.

**Table 5:** Summary of characteristics for the four latent profiles.

Profile label	Profile name	Defining characteristics	Sample size
C1	Low dependency-medium control	Low MPD scores across all dimensions; moderate self-regulation that maintains behavioral balance.	*n* = 109 (19.5%)
C2	High dependency-no control	Severe mobile dependency with high withdrawal; extremely low self-control and inability to resist digital temptations.	*n* = 207 (37.0%)
C3	No dependency-high control	Optimal profile with superior impulse regulation and resilience; minimal to no mobile phone dependency.	*n* = 191 (34.1%)
C4	Moderate dependency-low control	Moderate dependency with insufficient self-regulatory resources; prone to loss of control despite awareness.	*n* = 53 (9.5%)

To further verify the distinctiveness of the identified profiles, a series of Wald tests were conducted to compare the mean scores of the nine indicators across the four classes. As shown in Table 6, the results revealed significant differences among the four profiles on all dimensions of mobile phone dependency and self-control (all *F* > 120, *p* < 0.001). Post-hoc comparisons confirmed that each profile exhibited a unique psychological signature: for instance, Category C3 (No dependency-High control) showed significantly higher self-control and lower dependency scores than all other groups, while Category C2 (High dependency-No control) presented the most maladaptive pattern. These statistical differences provide strong evidence for the structural validity of the four-class solution.

**Table 6:** Mean comparison of MPD and SCS dimensions across the four latent profiles (*n* = 560).

Indicators	C1 (*n* = 109)	C2 (*n* = 207)	C3 (*n* = 191)	C4 (*n* = 53)	F/Wald χ^2^	*p*
MPD dimensions						
1. Withdrawal	2.15 ± 0.42	4.12 ± 0.51	1.52 ± 0.35	3.18 ± 0.48	156.42	<0.001
2. Loss of control	2.08 ± 0.39	4.05 ± 0.47	1.48 ± 0.32	3.05 ± 0.44	142.18	<0.001
3. Escapism	2.21 ± 0.45	3.98 ± 0.53	1.61 ± 0.38	3.12 ± 0.51	128.75	<0.001
4. Inefficiency	2.10 ± 0.40	3.85 ± 0.49	1.55 ± 0.34	2.95 ± 0.46	134.56	<0.001
SCS dimensions						
5. Impulse control	3.45 ± 0.52	1.92 ± 0.38	4.58 ± 0.45	2.65 ± 0.42	210.34	<0.001
6. Health habits	3.38 ± 0.48	1.85 ± 0.35	4.42 ± 0.42	2.58 ± 0.40	198.62	<0.001
7. Temptation resistance	3.52 ± 0.55	2.01 ± 0.41	4.65 ± 0.48	2.72 ± 0.45	185.27	<0.001
8. Focus on work	3.42 ± 0.50	1.95 ± 0.39	4.55 ± 0.44	2.60 ± 0.41	192.84	<0.001
9. Moderation in Leisure	3.35 ± 0.49	1.88 ± 0.36	4.48 ± 0.43	2.55 ± 0.39	204.15	<0.001

C1: Low dependency-Medium control; C2: High dependency-No control; C3: No dependency-High control; C4: Moderate dependency-Low control. Values are presented as Mean ± SD.

### 3.4 Comparison of mobile phone dependence and self-control latent categories among sub-health urban older adults with different demographic characteristics

Statistical results reveal that the latent categories of mobile phone dependency and self-control vary significantly across demographic groups within the sub-healthy urban older population. Specifically, significant disparities were observed based on gender, age, ethnicity, marital status, educational attainment, and monthly income (*p* < 0.001) (see Table 7 for details).

**Table 7:** Univariate analysis of latent profiles for mobile phone dependency and self-control among sub-healthy older adults in urban areas.

Indicator	Classification	Low dependency-medium control	High dependence-no control	No dependence-high control	Moderate dependency-low control	χ^2^	*P*
Gender	Male	78	101	99	1	69.713	<0.001
	Female	31	106	92	52		
Age	60–69	59	56	50	3	104.267	<0.001
	70–79	28	103	68	7		
	80 and above	22	48	73	43		
Ethnicity	Han	106	207	173	50	22.431	<0.001
	Other	3	0	18	3		
Marital status	Married	102	194	115	18	155.425	<0.001
	Unmarried	5	7	19	19		
	Other	2	6	57	16		
Educational attainment	Junior secondary school and below	71	49	34	3	475.100	<0.001
	Senior high school	29	100	61	3		
	University	9	50	89	0		
	Master’s degree and above	0	8	7	47		
Monthly income	Low income	57	53	62	3	111.762	<0.001
	Middle income	25	98	57	2		
	High income	27	56	72	48		

The four latent categories of mobile phone dependency and self-control served as the dependent variable. Statistically significant factors from the univariate analysis in Table 7 were incorporated as independent variables into a multiple logistic regression model. Findings indicate that gender, age, ethnicity, marital status, educational attainment, and monthly income are important predictors of category (all *p* < 0.05). These findings are detailed in Tables 8, 9.

**Table 8:** Multivariate analysis of mobile phone dependence and self-control in sub-health older urban residents.

Group	Indicator	B	Standard error	Wald	Degrees of freedom	Significance	Exp(B)	95% confidence interval for Exp(B)	
								Lower bound	Upper bound
2	Intercept	11.647	0.749	241.576	1	0.000			
	Gender	0.775	0.274	7.993	1	0.005	2.170	1.268	3.712
	Age	0.413	0.173	5.660	1	0.017	1.511	1.075	2.123
	Ethnic	−15.402	0.000	0.000	1	0.000	0.000	0.000	0.000
	Marital status	0.166	0.371	0.199	1	0.656	1.180	0.570	2.443
	Level of education	1.133	0.189	35.957	1	0.000	3.104	2.144	4.495
	Monthly income	0.232	0.168	1.899	1	0.168	1.261	0.907	1.754
3	Intercept	−9.038	1.283	49.647	1	0.000			
	Gender	0.613	0.303	4.093	1	0.043	1.846	1.019	3.343
	Age	0.694	0.192	13.046	1	0.000	2.003	1.374	2.919
	Ethnic	1.913	0.806	5.636	1	0.018	6.770	1.396	32.834
	Marital status	1.586	0.345	21.147	1	0.000	4.882	2.484	9.597
	Level of education	1.474	0.205	51.842	1	0.000	4.365	2.923	6.519
	Monthly income	0.355	0.186	3.639	1	0.056	1.426	0.990	2.053
4	Intercept	−30.935	4.086	57.319	1	0.000			
	Gender	4.715	1.312	12.918	1	0.000	111.599	8.532	1459.747
	Age	1.136	0.419	7.374	1	0.007	3.116	1.372	7.076
	Ethnic	3.713	1.293	8.249	1	0.004	40.966	3.251	516.152
	Marital status	1.900	0.446	18.133	1	0.000	6.685	2.788	16.029
	Level of education	3.336	0.395	71.357	1	0.000	28.116	12.965	60.974
	Monthly income	1.852	0.463	15.987	1	0.000	6.371	2.570	15.791

**Table 9:** Likelihood ratio test results for demographic factors of sub-health older population in urban areas.

Effect	Model fitting conditions	Likelihood ratio test		
	Log-likelihood of the simplified model	Chi-square	Degrees of freedom	Significance
Intercept	686.795	169.075	3	0.000
Gender	552.621	34.900	3	0.000
Age	533.324	15.603	3	0.001
Ethnicity	546.083	28.362	3	0.000
Marital status	601.843	84.123	3	0.000
Level of education	647.663	129.942	3	0.000
Monthly income	538.900	21.180	3	0.000

### 3.5 Relationship between mobile phone dependence and self-control in sub-health older urban residents and Tai Chi exercise

In the multivariate logistic regression model, the “Low dependency-Medium control” group was designated as the reference category to facilitate comparison with other latent profiles. We analyzed the relationship between Tai Chi exercise and the four latent categories of the OAMPDS-SCS. In this case, the latent categories were the dependent variable and Tai Chi exercise was the independent variable. Demographic variables—including gender, age, ethnicity, marital status, education, and income—were controlled. The “Low dependency-Medium control” category served as the reference group. Results indicate that Tai Chi exercise correlates negatively with both the “Moderate dependency-low control” and “High dependency-No control” categories (*p* < 0.05). This implies that individuals with lower Tai Chi participation are more likely to fall into these two groups. Conversely, Tai Chi exercise correlates positively with the “No dependency-High control” category (*p* < 0.05). Thus, individuals with higher physical exercise levels are significantly more likely to belong to this optimal category.

## 4 Discussion

The findings reveal that mobile phone dependency and self-control among sub-healthy older adults comprise four distinct types: Low dependency-Medium control, High dependency-No control, No dependency-High control, and Moderate dependency-Low control. Notably, High dependency-No control category accounted for 37.0% of all participants. The No dependency-High control category comprised 34.1%. This proportion is significantly higher than the other two categories. They also surpass findings from previous studies (36). Classification outcomes differ from prior research as well (37). This discrepancy likely stems from the simultaneous consideration of two variables in this study. This method produces a more granular segmentation of the latent profiles. The high prevalence of dependency indicates that the sub-healthy urban older adults cohort generally struggles with low self-control. They also exhibit poor health habit formation. This is closely associated with physiological and psychological changes characteristic of sub-health in old age (38). From a neurobiological perspective, the sensitivity of the reward system in the brains of urban sub-healthy older adults is often diminished. Consequently, they require prolonged mobile phone usage to achieve the same level of pleasure. This leads to a loss of control (39). The distribution of these profiles reveals group-specific demographic patterns. These insights are crucial for understanding intrinsic heterogeneity within this population. They also aid in developing precise intervention strategies.

On the gender dimension, older women have much higher proportions of “Moderate dependency-Low control” groups than men. This discrepancy may be related to women’s more nuanced and intense intrinsic needs for emotional expression and social connection in later life (40). Research shows that women are more likely to experience social circle contraction post retirement, leading them to rely on mobile phones to keep social interaction and emotional support. If self-regulation abilities do not adapt synchronously, this leads to heightened susceptibility to acquiring mobile phone dependency (41). This also suggests that when promoting group-based mind–body exercises such as Tai Chi, emphasis should be placed on their role in fulfilling and guiding the socio-emotional needs of older women.

Regarding age, individuals aged 70–79 comprise the largest proportion of the “High dependency-No control” group. This likely reflects a transitional phase in physical and mental function (42). They still have a certain willingness to explore new technologies like smartphones. However, partial physical decline increases their reliance on devices for accessing daily services. Additionally, the natural deterioration of the prefrontal cortex-linked to self-control-often occurs during this stage. Collectively, these factors make this subgroup the most vulnerable to dependency (43). Conversely, those aged 80 and above predominantly fall into the “non-dependent-high control” or “moderately dependent-low control” categories. This is attributed to fixed lifestyles and lower mobile phone acceptance. They also retain established behavioral self-regulation mechanisms (44). However, some older adults may develop dependency in specific scenarios due to health management needs, but without having systematic control (45).

On educational attainment, those with master’s degrees or higher present a significantly higher proportion in the “Moderate dependency-Low control” group. This reveals a contradictory pattern of “high cognition, low execution.” Advanced digital literacy facilitates extensive mobile utilization for academic exchange and smart health management. However, specific knowledge structures and social attributes may cause a cognitive-behavioral disconnect (46). Consequently, interventions for highly educated groups must extend beyond knowledge dissemination. The focus should shift to cultivating sound behavioral habits.

Regarding income, high-earners primarily populate the “High dependency-No control” and “No dependency-High control” groups. This suggests greater access to alternative recreational resources and premium wellness facilities. Objectively, this provides better conditions for self-regulation (47). However, professional or social continuity requirements may induce specific dependencies in some high earners (48, 49).

In summary, significant intra-group heterogeneity exists regarding mobile phone dependency and self-control among urban sub-healthy older adults. This diversity correlates strongly with gender, developmental stages, education, and socioeconomic resources. Future promotion of traditional exercises like Tai Chi must account for these variations. Interventions should respect the specific psychological traits and motivations of different subgroups. Implementing targeted facilitation strategies could improve physical and mental health while bolstering smartphone usage regulation.

The findings indicate a strong association between Tai Chi practice and the latent profiles of MPD and SC. Specifically, individuals with lower Tai Chi exercise frequency and shorter durations were more likely to be categorized into the “Moderate dependency-Low control” and “High dependency-No control” groups. Conversely, higher intensity and regularity of Tai Chi practice were significantly correlated with the “No dependency-High control” category. This suggests that Tai Chi activity serves as a significant correlate of these psychological characteristics. Older adults with less physical activity exhibit higher dependency and reduced self-regulation, which may be related to weaker psychological adaptation and limited self-regulatory resources (50). In contrast, regular practitioners demonstrate higher self-control and lower dependency tendencies, supporting the link between Tai Chi and enhanced self-regulatory capacity. Consistent with existing research, these findings suggest Tai Chi’s feasibility as a mind–body intervention (51). It is associated with mitigated excessive mobile use through the potential bolstering of self-control capabilities. Furthermore, long-term practice is linked to improved physical health, self-efficacy, and life satisfaction (52). Therefore, developing differentiated promotion strategies is essential. Tailoring interventions to specific MPD-SC profiles may help enhance self-regulation, reduce dependency risks, and facilitate a transition toward healthier states.

While the odds ratio for Tai Chi practice in certain categories remains high (OR = 111.599), this primarily reflects the exceptionally strong protective association between regular exercise and the “no dependency-high control” profile, rather than statistical artifacts. However, due to the relatively small sample size in specific subgroups, these magnitude estimates should be interpreted as indicative of a strong directional effect rather than a precise point estimate.

In addition, the current study qualitatively explored the potential moderating effects of gender and age on the relationship between Tai Chi and the identified latent profiles. The preliminary findings suggest that Tai Chi exercise may exert a universally positive impact regardless of gender or age within the sub-healthy older adults. However, due to the limited sample size in certain specific subgroups (e.g., C4), this study was not powered to detect complex interaction effects. Future research with larger, more diverse cohorts is warranted to conduct robust subgroup analyses and interaction testing.

## 5 Limitations

This study contains several limitations. Firstly, the cross-sectional design precludes causal inferences regarding MPD, self-control, and Tai Chi practice. Future longitudinal studies are necessary to address this gap. Second, the sample was restricted to sub-healthy residents of Xuzhou City, Jiangsu Province. This may introduce regional bias. Future research requires larger sample sizes and broader geographical coverage. Third, the study did not examine disease heterogeneity within the sub-healthy population. Future interventions should account for variables such as disease type, severity, medical expenses, and recurrence rates. Finally, the survey was limited to individuals aged 60 and above. Future stratified research in various age groups is likely to determine the turning points of dependency and self-control. This would facilitate targeted preventive measures at various life stages to optimize health status.

Moreover, One significant limitation is the potential selection bias, as all participants were recruited from established Tai Chi practice sites. This active population may already possess higher self-control or lower levels of mobile phone dependence than sedentary older adults. Consequently, the observed associations may be overestimated. Future studies should include a sedentary control group to further validate these findings.

## 6 Conclusion

Latent profile analysis (LPA) was employed in this study to determine four different subgroups among urban sub-healthy older adults. It revealed significant disparities in MPD and self-control levels within these groups. Demographic factors such as gender, age, ethnicity, marital status, educational attainment, and monthly income are key predictors of category membership. The findings confirm that Tai Chi, as a traditional mind–body exercise, fosters physical and mental well-being in this population. However, its efficacy varies across different subgroups. This discovery contributes to population-specific health behavior theory. It also provides empirical evidence for designing multi-tiered, individualized health promotion programs. Accurately identifying group characteristics allows for the precise utilization of Tai Chi. Ultimately, this approach improves sub-health status and strengthens behavioral regulation capabilities.

## Data Availability

The original contributions presented in the study are included in the article/Supplementary material, further inquiries can be directed to the corresponding author.
