# Supplementary material for: The relationship between mobile phone dependence, self-control, and Tai Chi exercise among sub-health older adults in urban areas: a latent profile analysis
Source: Front Public Health. 2026 Feb 11;14:1759896. doi: 10.3389/fpubh.2026.1759896 (PMC12932528; doi:10.3389/fpubh.2026.1759896)
Supplement: Supplementary file 1 [file Table_1.docx]

Supplementary Material

# 1 Supplementary Figures and Tables

Table 1. Correlation analysis between mobile phone dependency and self-control scale scores among sub-healthy elderly individuals in urban areas (r values, n=560)

| Variable | Mean | Standard Deviation | 1 | 2 | 3 | 4 | 5 | 6 | 7 | 8 | 9 |
| --- | --- | --- | --- | --- | --- | --- | --- | --- | --- | --- | --- |
| 1. Withdrawal | 2.931 | 1.087 | - |  |  |  |  |  |  |  |  |
| 2. Uncontrollability | 2.838 | 1.002 | 0.591** | - |  |  |  |  |  |  |  |
| 3. Avoidance | 2.862 | 1.044 | 0.584** | 0.545** | - |  |  |  |  |  |  |
| 4. Inefficiency | 2.827 | 1.005 | 0.547** | 0.494** | 0.526** | - |  |  |  |  |  |
| 5. Impulse Control | 3.031 | 1.093 | -0.632** | -0.514** | -0.526** | -0.472** | - |  |  |  |  |
| 6. Health Habits | 3.074 | 1.203 | -0.561** | -0.541** | -0.518** | -0.521** | 0.566** | - |  |  |  |
| 7. Resisting temptation | 3.099 | 1.2 | -0.600** | -0.572** | -0.547** | -0.509** | 0.552** | 0.521** | - |  |  |
| 8. Focus on work | 3.102 | 1.164 | -0.566** | -0.516** | -0.547** | -0.540** | 0.479** | 0.543** | 0.493** | - |  |
| 9. Restricting Entertainment | 3.069 | 1.162 | -0.506** | -0.505** | -0.505** | -0.495** | 0.491** | 0.475** | 0.526** | 0.535** | - |

Note. * p<0.05 ** p<0.01

Table 2. Model fit indices for latent profile models of mobile phone dependency and self-control among older adults

|  | AIC | BIC | aBIC | LMR(p) | BLRT(p) | Entropy | Grouping Situation |
| --- | --- | --- | --- | --- | --- | --- | --- |
| 1class | 15328.445 | 15,406.348 | 15349.207 | - | - | - | 560 |
| 2class | 12852.801 | 12,973.983 | 12,885.097 | 0.000 | 0.000 | 0.832 | 206, 354 |
| 3class | 12592.516 | 12756.977 | 12636.347 | 0.000 | 0.000 | 0.867 | 192, 210, 158 |
| 4class | 12565.221 | 12772.962 | 12620.586 | 0.001 | 0.000 | 0.875 | 109, 207, 191, 53 |

Table 3 Univariate analysis of latent profiles for mobile phone dependency and self-control among sub-healthy elderly individuals in urban areas

| Indicator | Classification | Low Dependence-Moderate Control | High Dependence-No Control | No Dependence-High Control | Medium Dependency- Low Control | X^2^ | P |
| --- | --- | --- | --- | --- | --- | --- | --- |
| Gender | Male | 78 | 101 | 99 | 1 | 69.713 | <0.001 |
|  | Female | 31 | 106 | 92 | 52 |  |  |
| Age | 60–69 | 59 | 56 | 50 | 3 | 104.267 | <0.001 |
|  | 70–79 | 28 | 103 | 68 | 7 |  |  |
|  | 80 and above | 22 | 48 | 73 | 43 |  |  |
| Ethnicity | Han | 106 | 207 | 173 | 50 | 22.431 | <0.001 |
|  | Other | 3 | 0 | 18 | 3 |  |  |
| Marital Status | Married | 102 | 194 | 115 | 18 | 155.425 | <0.001 |
|  | Unmarried | 5 | 7 | 19 | 19 |  |  |
|  | Other | 2 | 6 | 57 | 16 |  |  |
| Educational attainment | Junior secondary school and below | 71 | 49 | 34 | 3 | 475.100 | <0.001 |
|  | Senior High School | 29 | 100 | 61 | 3 |  |  |
|  | University | 9 | 50 | 89 | 0 |  |  |
|  | Master's degree and above | 0 | 8 | 7 | 47 |  |  |
| Monthly income | Low income | 57 | 53 | 62 | 3 | 111.762 | <0.001 |
|  | Middle income | 25 | 98 | 57 | 2 |  |  |
|  | High income | 27 | 56 | 72 | 48 |  |  |

Table 4 Multivariate Analysis of Mobile Phone Dependence and Self-Control in Sub-Health Elderly Urban Residents

| Group | Indicator | B | Standard Error | Wald | Degrees of freedom | Significance | Exp(B) | 95% confidence interval for Exp(B) | |
| --- | --- | --- | --- | --- | --- | --- | --- | --- | --- |
|  |  |  |  |  |  |  |  | Lower Bound | Upper Bound |
| 2 | Intercept | 11.647 | 0.749 | 241.576 | 1 | 0.000 |  |  |  |
|  | Gender | 0.775 | 0.274 | 7.993 | 1 | 0.005 | 2.170 | 1.268 | 3.712 |
|  | Age | 0.413 | 0.173 | 5.660 | 1 | 0.017 | 1.511 | 1.075 | 2.123 |
|  | Ethnic | -15.402 | 0.000 | 0.000 | 1 | 0.000 | 0.000 | 0.000 | 0.000 |
|  | Marital status | 0.166 | 0.371 | 0.199 | 1 | 0.656 | 1.180 | 0.570 | 2.443 |
|  | Level of education | 1.133 | 0.189 | 35.957 | 1 | 0.000 | 3.104 | 2.144 | 4.495 |
|  | Monthly income | 0.232 | 0.168 | 1.899 | 1 | 0.168 | 1.261 | 0.907 | 1.754 |
| 3 | Intercept | -9.038 | 1.283 | 49.647 | 1 | 0.000 |  |  |  |
|  | Gender | 0.613 | 0.303 | 4.093 | 1 | 0.043 | 1.846 | 1.019 | 3.343 |
|  | Age | 0.694 | 0.192 | 13.046 | 1 | 0.000 | 2.003 | 1.374 | 2.919 |
|  | Ethnic | 1.913 | 0.806 | 5.636 | 1 | 0.018 | 6.770 | 1.396 | 32.834 |
|  | Marital status | 1.586 | 0.345 | 21.147 | 1 | 0.000 | 4.882 | 2.484 | 9.597 |
|  | Level of education | 1.474 | 0.205 | 51.842 | 1 | 0.000 | 4.365 | 2.923 | 6.519 |
|  | Monthly income | 0.355 | 0.186 | 3.639 | 1 | 0.056 | 1.426 | 0.990 | 2.053 |
| 4 | Intercept | -30.935 | 4.086 | 57.319 | 1 | 0.000 |  |  |  |
|  | Gender | 4.715 | 1.312 | 12.918 | 1 | 0.000 | 111.599 | 8.532 | 1459.747 |
|  | Age | 1.136 | 0.419 | 7.374 | 1 | 0.007 | 3.116 | 1.372 | 7.076 |
|  | Ethnic | 3.713 | 1.293 | 8.249 | 1 | 0.004 | 40.966 | 3.251 | 516.152 |
|  | Marital status | 1.900 | 0.446 | 18.133 | 1 | 0.000 | 6.685 | 2.788 | 16.029 |
|  | Level of education | 3.336 | 0.395 | 71.357 | 1 | 0.000 | 28.116 | 12.965 | 60.974 |
|  | Monthly income | 1.852 | 0.463 | 15.987 | 1 | 0.000 | 6.371 | 2.570 | 15.791 |

Table 5 Likelihood Ratio Test Results for Demographic Factors of Sub-Health Elderly Population in Urban Areas

| Effect | Model Fitting Conditions | Likelihood Ratio Test | | |
| --- | --- | --- | --- | --- |
|  | Log-likelihood of the simplified model | Chi-Square | Degrees of freedom | Significance |
| Intercept | 686.795 | 169.075 | 3 | 0.000 |
| Gender | 552.621 | 34.900 | 3 | 0.000 |
| Age | 533.324 | 15.603 | 3 | 0.001 |
| Ethnicity | 546.083 | 28.362 | 3 | 0.000 |
| Marital Status | 601.843 | 84.123 | 3 | 0.000 |
| Level of Education | 647.663 | 129.942 | 3 | 0.000 |
| Monthly income | 538.900 | 21.180 | 3 | 0.000 |


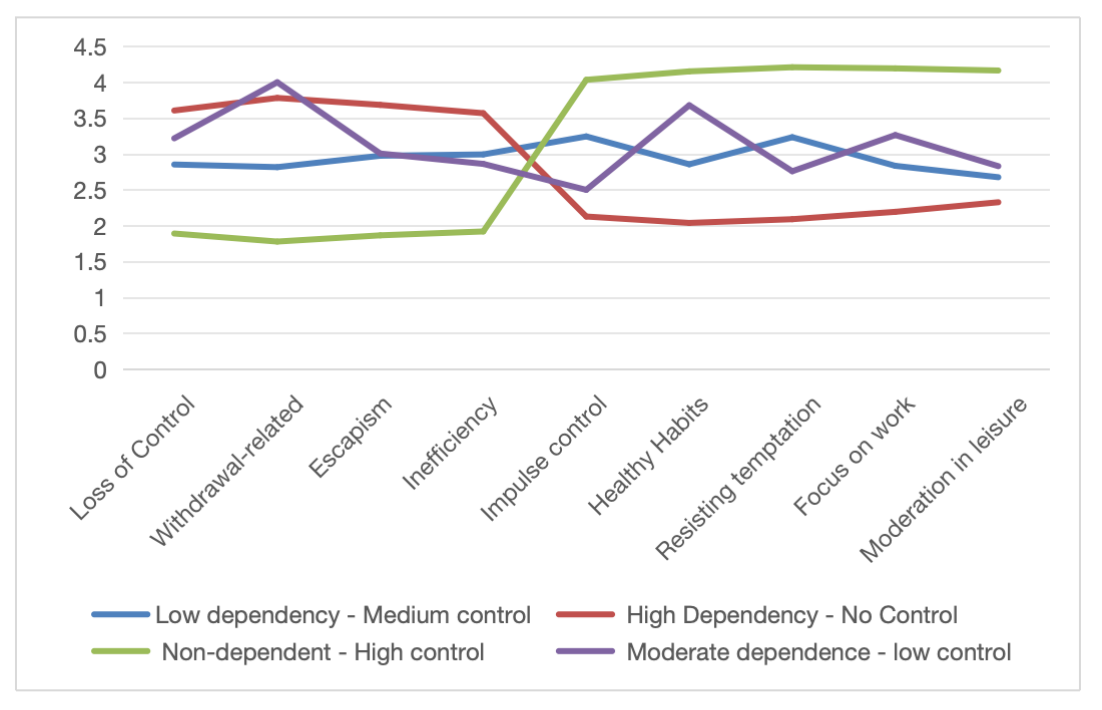


Figure 1. Scores of MPAI and SCS Dimensions for Latent Categories of Mobile Phone Dependence and Self-Control in Older Adults
